# FluShuffle and FluResort: new algorithms to identify reassorted strains of the influenza virus by mass spectrometry

**DOI:** 10.1186/1471-2105-13-208

**Published:** 2012-08-20

**Authors:** Aaron TL Lun, Jason WH Wong, Kevin M Downard

**Affiliations:** 1 School of Molecular Bioscience G-08, The University of Sydney, Sydney, NSW, 2006, Australia; 2 Prince of Wales Clinical School and Lowy Cancer Research Centre, University of New South Wales, Sydney, NSW, Australia

**Keywords:** Influenza virus, Reassortment, Proteotyping, Computer algorithm, Phylogenetics, Mass spectrometry

## Abstract

**Background:**

Influenza is one of the oldest and deadliest infectious diseases known to man. Reassorted strains of the virus pose the greatest risk to both human and animal health and have been associated with all pandemics of the past century, with the possible exception of the 1918 pandemic, resulting in tens of millions of deaths. We have developed and tested new computer algorithms, FluShuffle and FluResort, which enable reassorted viruses to be identified by the most rapid and direct means possible. These algorithms enable reassorted influenza, and other, viruses to be rapidly identified to allow prevention strategies and treatments to be more efficiently implemented.

**Results:**

The FluShuffle and FluResort algorithms were tested with both experimental and simulated mass spectra of whole virus digests. FluShuffle considers different combinations of viral protein identities that match the mass spectral data using a Gibbs sampling algorithm employing a mixed protein Markov chain Monte Carlo (MCMC) method. FluResort utilizes those identities to calculate the weighted distance of each across two or more different phylogenetic trees constructed through viral protein sequence alignments. Each weighted mean distance value is normalized by conversion to a Z-score to establish a reassorted strain.

**Conclusions:**

The new FluShuffle and FluResort algorithms can correctly identify the origins of influenza viral proteins and the number of reassortment events required to produce the strains from the high resolution mass spectral data of whole virus proteolytic digestions. This has been demonstrated in the case of constructed vaccine strains as well as common human seasonal strains of the virus. The algorithms significantly improve the capability of the proteotyping approach to identify reassorted viruses that pose the greatest pandemic risk.

## Background

Influenza is one of the oldest and deadliest infectious diseases known to man. Seasonal human influenza epidemics are responsible for over 250,000 deaths worldwide and over 3 million cases of severe illness each year [1-3]. When a host is simultaneously infected with two or more strains derived from different animal species, reassortment events can occur producing progeny viruses that contain genes derived from two or more parent strains. This significantly changes a virus’ antigenic profile. It poses serious epidemiological consequences [4,5] due to a lack of host immunity against such novel strains particularly when one of the parent strains has been derived from an animal host, usually avian or swine [6-8].

Of the four influenza pandemics of the past century [9], at least three have been shown to be associated with reassorted strains. Reassortment among avian and human type A influenza viruses produced novel H2N2 and H3N2 strains that caused global human pandemics in 1957 and 1968 respectively [10,11]. The type A H1N1 swine-originating influenza virus associated with the 2009 pandemic was produced by a reassortant between a Eurasian swine virus and a triple reassortant North American swine virus of avian, human and swine origin [12]. Collectively, these pandemics have been associated with tens of million deaths worldwide. The rapid identification of reassorted strains of the virus is therefore an important requirement to mitigate the impact of influenza pandemics.

The most conventional method to identify reassorted influenza viruses involves the construction of phylogenetic trees based on the alignment of gene sequences for each viral protein [13]. Genes are first sequenced using the reverse transcriptase polymerase chain reaction (RT-PCR) [14]. Multiple sequence alignments for each gene segment are then performed using algorithms such as ClustalW [15]. Phylogenetic trees are then constructed based upon these alignments. Where different gene segments of a common strain are in conflicting position across the trees, a potential reassorted virus is identified.

Given that full gene sequencing [16] of a large number of strains is very time consuming, even with the advent of real time parallel PCR sequencing methods [17,18] and that multiple sequence alignments of full gene sequences are both computationally and time intensive, this approach has its limitations. As tree construction for all eight gene segments of the viral RNA is subsequently needed to establish a potential reassorted strain [19] algorithms have been developed to automate this process [20]. Rabadan and co-workers measured the Hamming distance between respective gene segments to establish the presence or absence of reassortment [21], while Nagarajan and Kingsford [22] considered distributions of phylogenetic trees for each gene segment rather than a single consensus tree. Others have pursued reassortment identification based on distance measurement using a complete composition vector (CCV) and segment clustering using a minimum spanning tree (MST) algorithm [23]. Considerations of only a quartet of trees at a time [24] and the use of reassortment networks [25] have also been employed to identify reassorted influenza viruses.

There remain advantages to studying protein over gene sequences for monitoring influenza strains and establishing reassortment [26] due to the degeneracy of the genetic code. Changes to the nucleotide bases at the third codon position provide little or no evolutionary information. Proteins provide a stronger phylogenetic signal associated with 20 possible amino acids at each sequence position versus just 4 nucleotides in the case of gene sequences [26]. The analysis of viral proteins by mass spectrometry is also more rapid and direct than the steps required to both amplify and sequence viral RNA by RT-PCR.

We have recently developed a new rapid and direct proteotyping approach with which to characterize the influenza virus [27-30]. Briefly, whole virus proteolytic digests are analysed by high resolution mass spectrometry to detect signature peptides that are conserved in sequence and unique in mass. These enable the type, subtype and lineage [31] of strains to be unambiguously identified without sequencing of the viral proteins, either in full or in part. A computer algorithm has been written to achieve this in an automated manner [32]. The approach can differentiate seasonal and pandemic H1N1 influenza viruses [33], identify the gene origin of reassorted strains [34] and has been used to study the evolution of H5N1 viruses [35]. The use of mass spectrometry in the proteotyping approach allows for the analysis of hundreds of virus digests at a rate of less than one minute per sample, even without human intervention on some automated instruments. The approach is limited only the time required for whole virus or protein digestion.

Here two new algorithms, known as FluShuffle and FluResort, are described which have been specifically written to identify reassortant influenza viruses from such data sets. FluShuffle considers different combinations of viral protein identities that match the mass spectral data using a Gibbs sampling algorithm. FluResort maps those identities onto phylogenetic trees, constructed through viral protein sequence alignments, to calculate the weighted distance of each across two or more different trees. Each weighted mean distance value is normalized by conversion to a Z-score that is used to establish the probability of a reassorted strain.

## Implementation

### Software design and development

**Figure 1:**
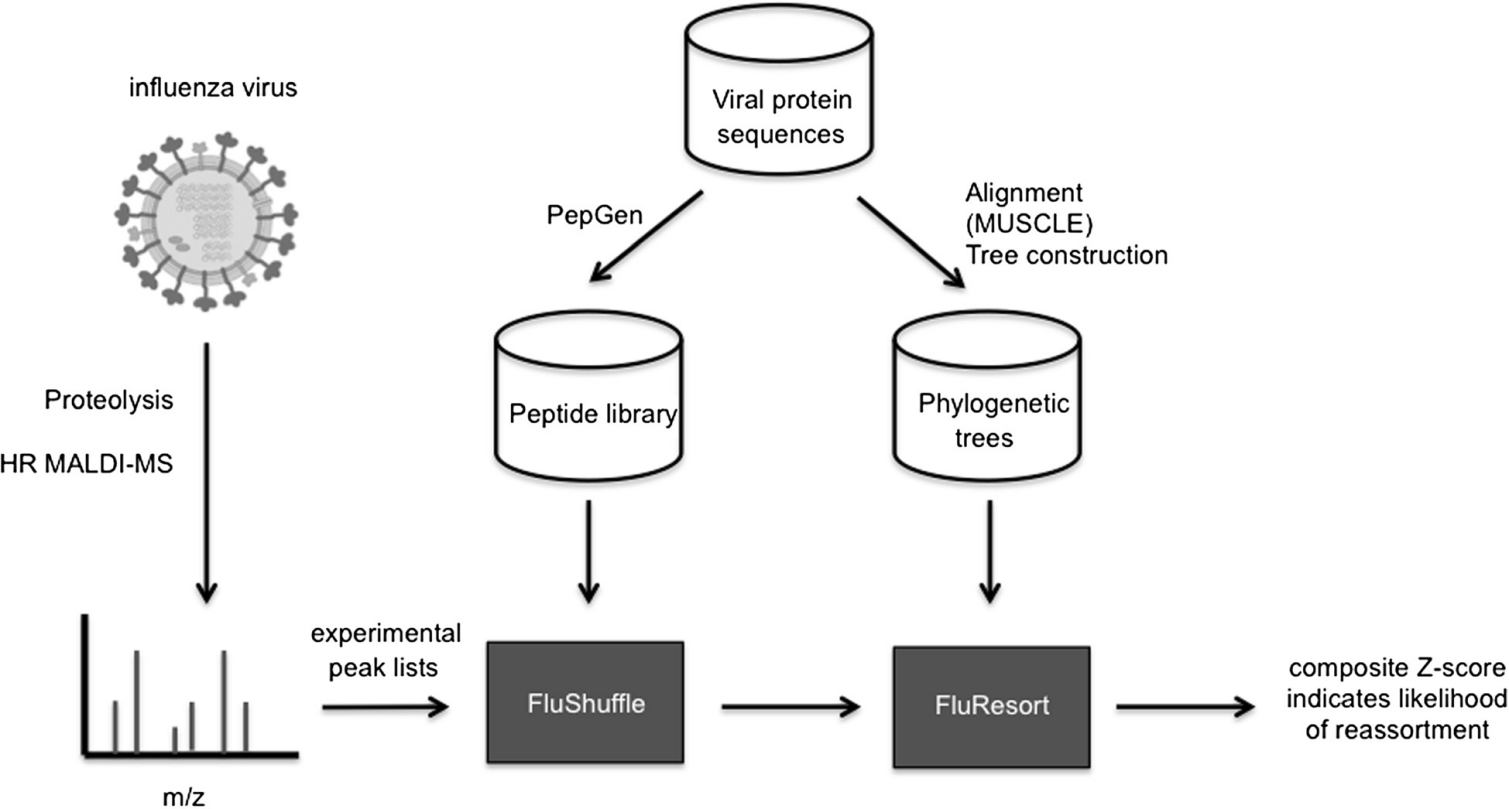
**An overview of the computational strategy and algorithms used to establish viral protein identity and reassorted strains.** The algorithms FluShuffle and FluResort are shaded.

The overall computational approach is shown in Figure 1 where the FluShuffle and FluResort algorithms are highlighted. Some auxiliary programs that were written for data manipulation prior to analysis are also shown. All programs were written in ANSI/ISO standard C++ and tested on Pentium4 and Intel i5 personal computers, with between 1–4 GB of RAM, running either the Microsoft Windows 7 or Kubuntu Linux 11.04 operating system. The FluShuffle and FluResort algorithms have been implemented to run via a web interface.

#### Theoretical peptide library preparation with PepGen

Viral protein sequence data derived from the NCBI Influenza Virus Resource [36], and those sequences representing common contaminant proteins in egg and cell culture grown viruses from the UniProt database were obtained in FASTA format. An algorithm, PepGen was developed to generate theoretical peptide monoisotopic masses with the protein accessions for each protein in the database. To achieve this, PepGen performs an *in silico* proteolytic digest of all non-redundant complete sequences was performed based on the specificity of trypsin or Glu-C endoproteinases (D and E cleavage). Autolysis products for these enzymes were also included. Peptides resulting from N-terminal post-translational cleavage were included in this dataset while all peptides with unknown residues were discarded. The theoretical monoisotopic mass was calculated for each protonated peptide ion [M + H]^+^ with and without methionine oxidation, N-terminal pyroglutamate formation and cysteine carbamidomethylation.

#### Viral protein identification with FluShuffle

The assignment of peaks in a mass spectrum consisting of a mixture of viral proteins is not trivial. A simple naive approach, where the distribution for each protein is estimated separately, will fail to account for the possibility that the peaks may originate from other proteins. This leads to incorrect assignments [37]. FluShuffle implements a Bayesian Markov Chain Monte Carlo (MCMC) approach [38] to assign a combination of protein accessions (one per viral protein) to a single mass spectrum.

The posterior probability for any given combination of proteins, *θ*, is the probability that the combination is present in the sample given the mass spectral data *D*. The expression for the posterior probability in equation 1 is a modified version of the posterior function presented in the ProFound algorithm [39].

(1) $$P\left(\theta |D\right)\propto P\left(\theta \right)\frac{\left(N-r\right)!}{\left(N+1\right)!\left(w+1\right)}\left(\prod_{i=1}^{r}\frac{a_{i}\Delta M}{2\sigma_{i}}\right)$$

*r* is the number of peaks in the spectrum that were matched to the set of theoretical peptides from all accessions in *θ*. *w* is the number of unmatched peaks. *N* Is the total number of theoretical peptides produced from digestion of all proteins in *θ*. α_*i*_ is the number of theoretical peptides matching to peak *i*. *σ*_*i*_ is the maximum mass error for peak *i* in Daltons. *ΔM* is the mass acquisition range. The posterior probability $P\left(\theta |D\right)$ can then described as a product of the prior (*P*(*θ*)) and likelihood functions according to Bayes’ theorem.

Note from equation 1, that as r increases (N-r)! will decrease. However, this is offset by the probability of a random peak. For two possible accessions, A and B, if A has a one extra peptide which matches to an observed peak, r increases by 1 and the value of (N-r)! decreases. However, an increase in r results in one more product to multiply within the ‘$\mathrm{a\Delta M}/\left(2\sigma_{i}\right)$’ term. For high resolution, high mass accuracy mass spectrometry, the term *σ*_*i*_ is very small. This results in a large ‘$\mathrm{a\Delta M}/\left(2\sigma_{i}\right)$’ term that will more than offset the decrease in the posterior probability resulting from a decrease in (N-r).

Larger values of the posterior probability represent an improved fit to the observed data. Increasing the number of matched peaks and decreasing the number of unmatched peaks will increase the posterior probability. The prior probability enables information about the strain to be included in the identification process. For example, the expected similarity between influenza strains of consecutive seasons can be used to define priors such that viral protein accessions from the previous season have a higher prior probability. The default priors are the historical frequencies of each accession in the database.

FluShuffle uses a Gibbs sampling algorithm to estimate the marginal posterior probability for each known accession. The marginal posterior probability simply represents the probability of that accession being present in the sample given the data. A higher probability indicates that there is more evidence for the presence of that particular accession. The Gibbs sampler is chosen as it can handle many parameters (i.e. proteins) simultaneously.

The Gibbs sampler algorithm generates a new combination of accessions at each iteration step. Let the combination of accessions be described by {s_1_, s_2_, … s_n_} where s_i_ is the current accession for protein *i*. At a new iteration, each accession in the database for protein 1 is combined with {s_2_, … s_n_} to generate a combination that is supplied to equation 1. This produces a conditional probability (as it is based on given values for the other proteins) for each accession of protein 1. A new accession is then randomly chosen with probability of selection equal to the conditional posterior probability for each accession. This new accession is used to replace *S*_1_ in the combination. This process is repeated for protein 2 except that each accession for protein 2 is combined with the updated accession for protein 1 as well as before calculation of the conditional posterior*.* More generally, the conditional posterior is calculated for each accession of protein *i* based on the updated values for proteins 1,2,…,*i*-1 and the existing values for proteins *i* + 1, *i* + 2, …n. The iteration is complete when the accession for each protein is updated.

FluShuffle repeats the process of step generation for a user-defined number of iterations (default is 5000) to produce a “chain” of steps. The Gibbs sampler is designed such that steps to high probability solutions are more likely to be generated than steps to low probability solutions. To avoid a biased posterior estimate, the first 10% of steps were discarded as burn-in. The MCMC algorithm nominates a starting combination of viral proteins (randomly or otherwise) and traverses the solution landscape by switching proteins to identify higher posterior probability solutions. In theory, if the algorithm were to run indefinitely, it doesn't matter if the starting combination is a low probability solution. In practice, due to run time limitations and computing power, there is some bias afforded by the starting combination that may never be visited during the rest of the run. To remove this bias, the first 10% of the steps of the run were discarded.

The remaining steps were used to estimate the posterior probability for each accession based on the proportion of steps that contained that accession. Accessions that match more peaks or match uniquely to a peak will be selected more often at each step in the Gibbs sampler as they have a higher conditional posterior probability. This means that they will be present in more steps and will have a higher posterior probability estimate.

#### Determination of virus reassortment with FluResort

Once the identity of the proteins arising from the virus digest has been established by FluShuffle, these identities are used to establish whether the virus is a reassorted strain. To facilitate this, the FluResort algorithm was developed. To establish a statistical model for the likelihood that a virus has been reassorted, the phylogenetic relationship between the strain of each identified protein and all other strains must be determined. To this end, the patristic distance derived from the phylogenetic tree of each viral protein was used.

For each accession *i*, FluResort calculates the patristic distance *d* to an observed protein accession from FluShuffle *j* using the sum of branch lengths from *i* to *j* in the phylogenetic tree. The distance is then weighted by the posterior probability of *p*_*j*_ in order to account for uncertainty from FluShuffle identification. Since FluShuffle will typically identify multiple candidate accessions with varying posterior probability, the weighted mean distance (i.e. the disparity between the proposed strain identity and the observed accessions), *x*_*i*_ is expressed as,

(2) $${\bar{x}}_{l}=\frac{\sum_{j\in K}p_{j}d_{ij}}{size\left(K\right)}$$

such that *K* is the set of all accessions identified by FluShuffle.

The variance of the weighted distances, $\sigma_{i}^{2}$_,_ is estimated using equation 3. This represents the uncertainty of disparity between each accession and the observed accessions. Uncertainty results in a large spread of distances as the observed accessions are spread throughout the tree. This results in a large variance.

(3) $$\sigma_{i}^{2}=\sum_{j\in K}pj{\left(d_{\mathrm{ij}}-{\bar{x}}_{i}\right)}^{2}$$

Each weighted mean distance value calculated by the FluResort algorithm was converted to a Z-score (equation 5). The accession with the lowest weighted mean distance, ${\bar{x}}_{0}$, was determined for each protein. The variance of the weighted distances, $\sigma_{0}^{2}$, was calculated for that accession using equation 3. This allows calculation of the Z-score, *Z*_*i*_, for the accession *i* corresponding to each strain based on its weighted mean distance, ${\bar{x}}_{i}$.

(4) $$Z_{i}=\frac{{\bar{x}}_{i}-{\bar{x}}_{0}}{\sigma_{0}}$$

The Z-score represents the fit of the proposed strain to the observed accessions. A higher Z-score corresponds to a poorer fit i.e. more evidence against the proposed strain. The difference between the protein accession and the lowest weighted mean distance, ${\bar{x}}_{0}$, is used as the numerator to guarantee that the lowest Z-score for each protein is zero. The variance is used as the denominator to account for uncertainty in protein identification. Confidence in the proposed strain is reduced as the uncertainty in protein identification increases. This is reflected in the formulation of the Z-score whereby larger variances will result in lower Z-scores.

The Z-scores for all accessions corresponding to a strain are then summed to provide the composite Z-score, *c*, for that strain. The “best-fitting” strain is that with the lowest composite Z-score. This can be repeated for combinations of strains where each strain contributes a complementary subset of proteins. A combination of two strains represents a reassorted virus from one reassortment event, a combination of three strains represents a reassorted virus from two reassortment events and so on. Minimum composite Z-scores were compared across differing numbers of reassortment events to determine whether or not the virus was reassorted. The decrease in composite Z-scores with increasing reassortment number must be large enough to justify an increase in the number of parameters. This was assessed using the standard deviation of the composite Z-scores (equation 5).

(5) $$\sigma_{c}=\sqrt{N}$$

The variance of the composite Z-score is equal to the sum of variances for each Z-score contributing to the composite. As each variance is normalized to 1, the sum of variances is equal to the number of proteins, *N*, being examined assuming that the identity of each viral protein is independent of one another. The standard deviation of the composite, *σ*_*c*_, can then be calculated as a function of *N*. A large difference in composite Z-scores was considered to be equal to or greater than $2\sqrt{N}$ (i.e. two standard deviations). This corresponds approximately to a 0.05 significance level for a two-tailed Z-test if the weighted distances are assumed to be normally distributed given the Z-score has an equivalent formulation to the Z-statistic.

## Results and discussion

### Application of FluShuffle and FluResort algorithms to analyze MS data of reassorted pandemic strain

The FluShuffle and FluResort algorithms were first tested with mass spectral data obtained from the digestion of a type A H1N1 strain produced from the reassortment of a 2009 H1N1 pandemic strain (A/California/07/2009) and a lab-modified H1N1 strain (A/Puerto Rico/08/1934). It was produced for a vaccine (PanVax 2009) against the 2009 H1N1 pandemic swine-originating influenza virus (SOIV) strains and retains the surface viral proteins, hemagglutinin and neuraminidase, of the pandemic strain to elicit an immune response against the native strain.

**Figure 2:**
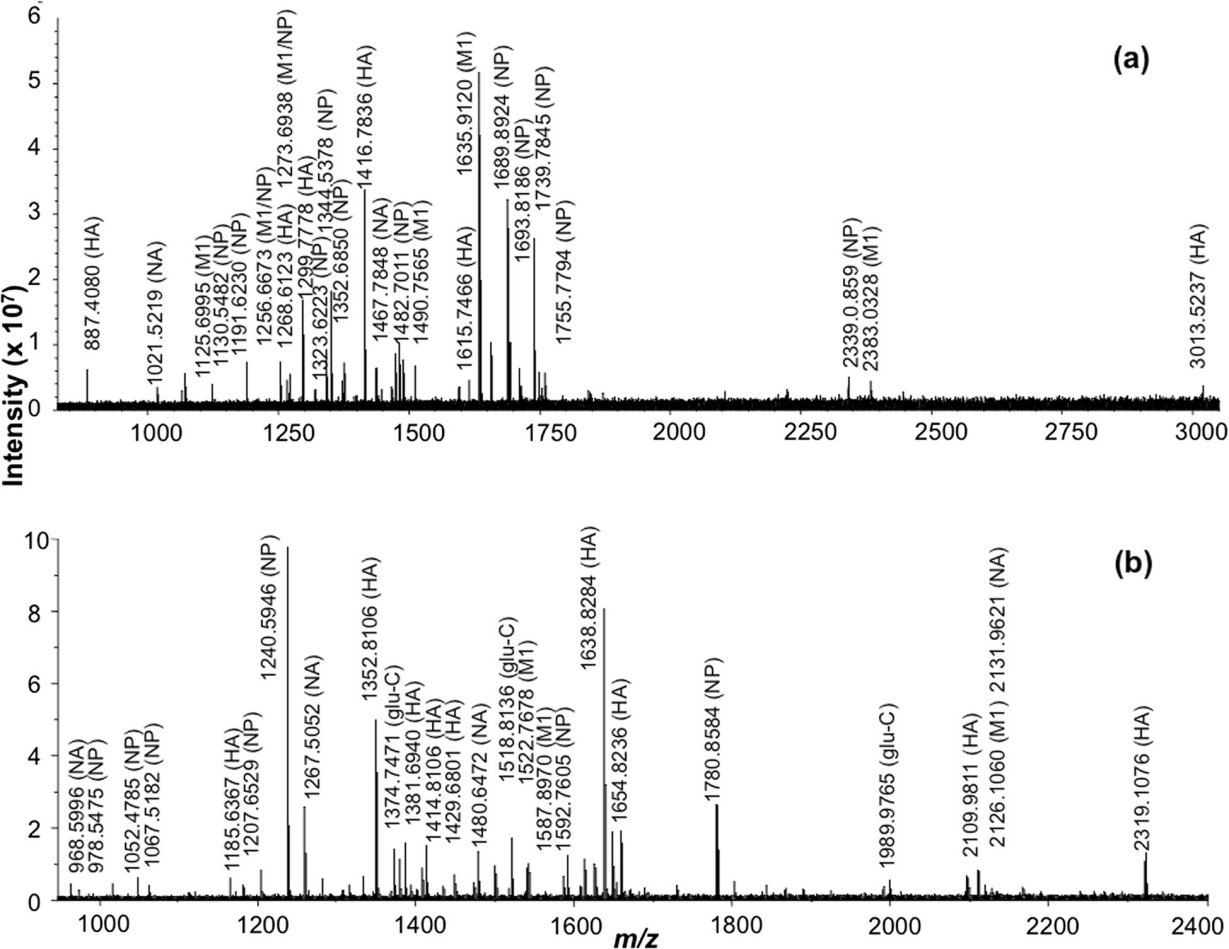
**High resolution MALDI mass spectrum of the (a) tryptic and (b) Glu-C endoproteinase whole virus digest of the PanVax vaccine against the 2009 H1N1 influenza pandemic strains.** Peaks labelled Glu-C denote autolysis products.

The FluShuffle algorithm was first used to perform a combined analysis on the high resolution MALDI mass spectra obtained from the respective whole virus digests of PanVax using trypsin and Glu-C endoproteinases (Figure 2). Monoisotopic mass values for 46 protonated peptide ions were identified in the mass spectrum resulting from the tryptic digest after signal-to-noise filtering and deisotoping (Figure 2a). Of these, 6 peptide ions were each matched to the hemagglutinin (HA) and matrix M1 proteins (Table 1) by FluShuffle. The nucleoprotein (NP) was matched to 13 ions whereas the neuraminidase (NA) protein matched 2 peptide ions. A further 2 peptide ions were matched to peptides from both M1 and NP that could not be distinguished due to their similar theoretical mass values.

**Table 1:** Posterior probabilities for the major viral proteins detected in the mass spectra of the whole virus digest mass spectra of the PanVax vaccine strain

**Viral antigen**	**Strain**	**Number of matched tryptic peptides**	**Assignment confidence (%) trypsin digest only**	**Number of matched Glu-C peptides**	**Assignment confidence (%) Glu-C digest only**	**Assignment confidence (%) combined digest**
HA	A/California/07/2009	6	100	9	100	100
NA	A/California/07/2009	2	46	3	100	100
NP	A/Puerto Rico/08/1934	13	100	7	30	100
M1	A/Puerto Rico/08/1934	6	47	3	1	66

Values are shown in percentages were estimated with FluShuffle and summed over the clade.

Monoisotopic mass values for 77 ion peaks were identified in the mass spectrum resulting from whole virus digestion with endoproteinase Glu-C (Figure 2b). Most of the high intensity ions in the mass spectrum were identified as being derived from viral proteins. Segments of the HA and NP proteins were matched to 9 and 7 ions respectively (Table 1). The NA and M1 proteins were matched to 3 ions each.

Other viral proteins matched to fewer than 2 mass values in each spectrum. This resulted in low confidences for each predicted identity. The low number of matches is associated with the low copy numbers of the polymerase subunits, non-structural (NS) proteins and M2 protein within each virion. Smaller proteins like M2 and NS2 are also less likely to be detected, as they contain fewer tryptic peptides for matching.

**Figure 3:**
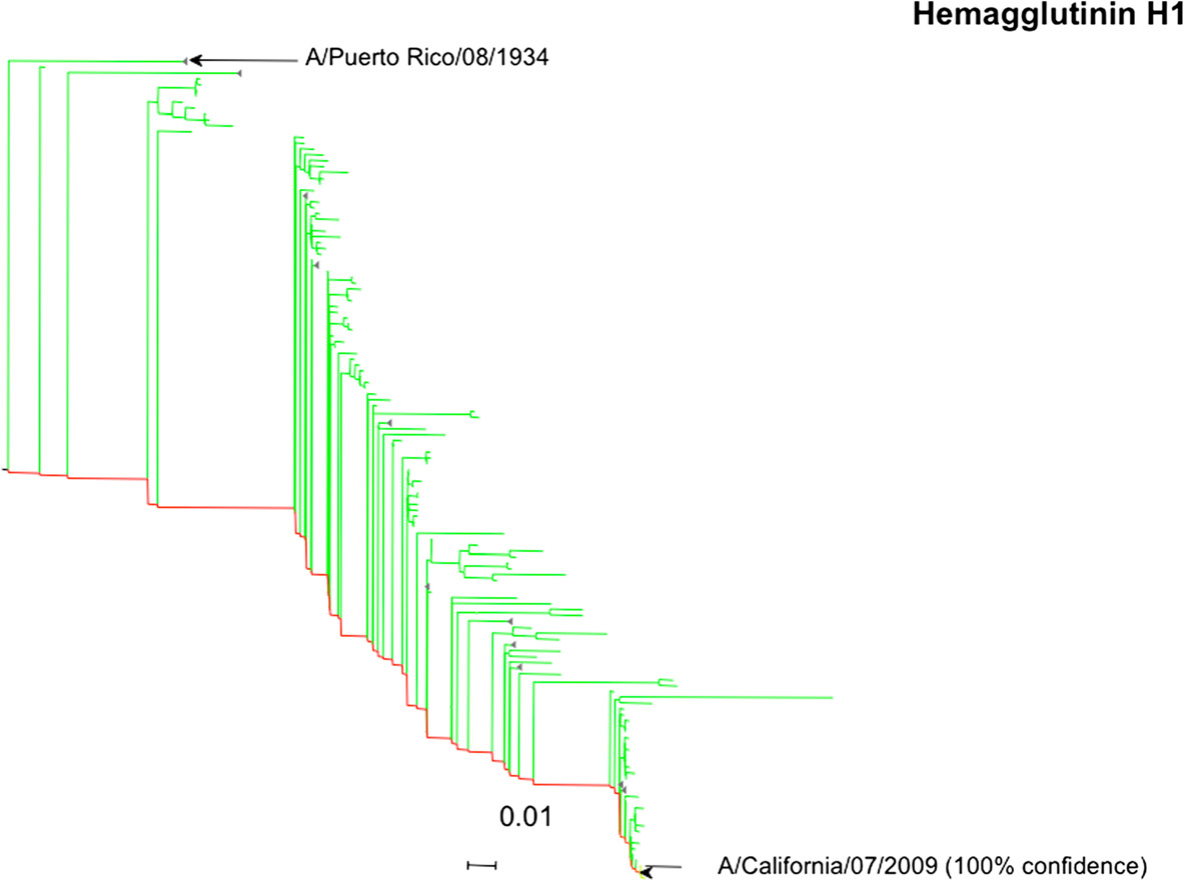
**Phylogenetic tree for the hemagglutinin protein (H1 subtype) with colouration of its predicted identity within the PanVax strain.** Irrelevant clades have been collapsed for clarity. A scale bar is shown that represents distance as substitutions per site. The location of the expected strain origin (A/California/07/2009) is labelled and the sum of probabilities for its clade of close relatives is shown in brackets as a percentage. The location of the A/Puerto Rico/08/1934 strain is also labelled.

The predicted protein identities obtained from the mass spectrum by FluShuffle were visualised by edge colouration in the mid-point rooted phylogenetic tree for each protein. The HA protein was clearly sub typed as H1 (Figure 3) with the origin further localised to the clade containing the A/California/07/2009 strain and its close relatives with 100% certainty. This is consistent with its identity origin in the PanVax strain. The same result was observed for the neuraminidase protein (see Additional file 1: Figure S1).

**Figure 4:**
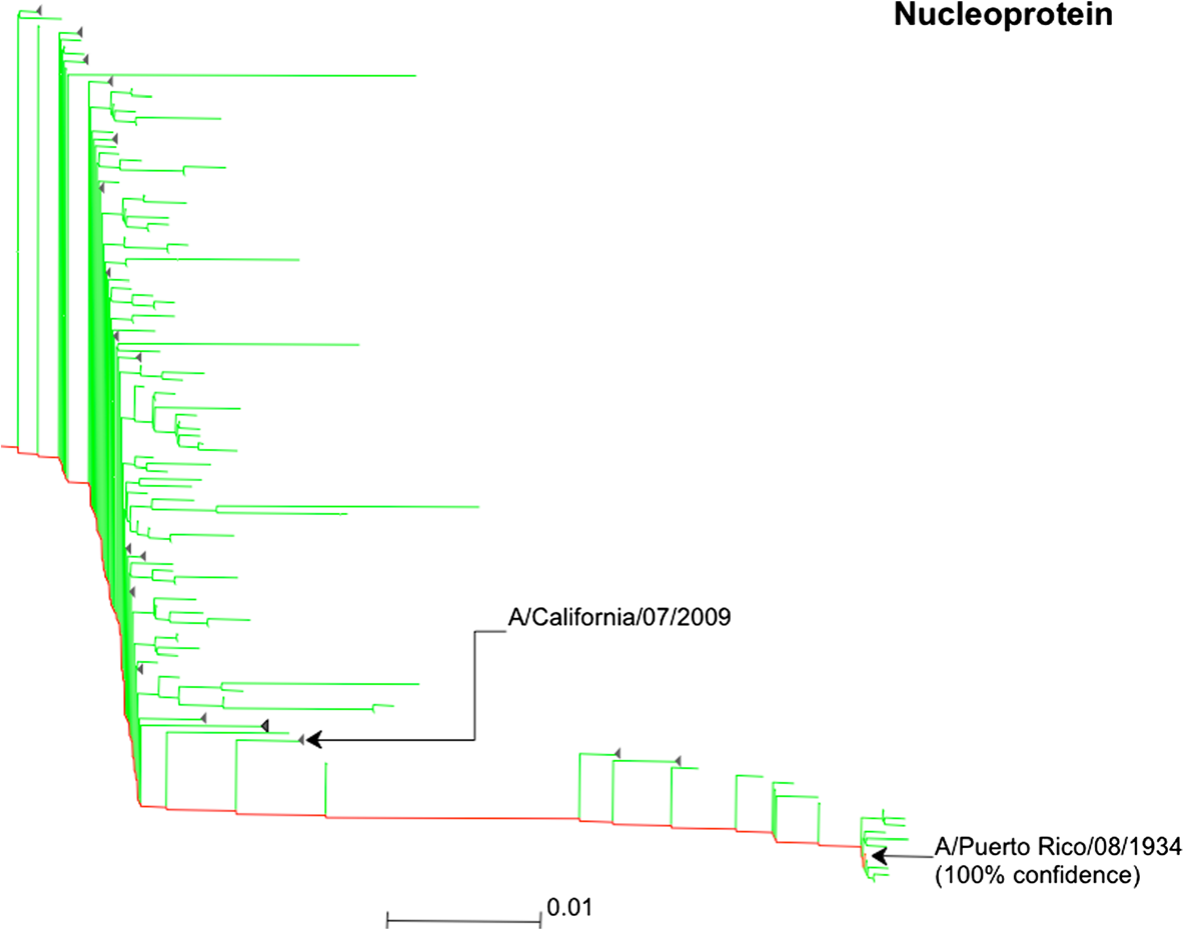
**Phylogenetic tree for the nucleoprotein for influenza type A with colouration of its predicted identity within the PanVax strain.** The location of the expected origin (A/Puerto Rico/08/1934) is labelled and the sum of probabilities for its clade of close relatives is shown in brackets as a percentage. The location of the A/California/07/2009 strain is also labelled.

The nucleoprotein was clearly identified as originating from a type A strain (Figure 4). Its predicted identity was localised to a clade containing close relatives of the A/Puerto Rico/08/1934 strain with 100% certainty consistent with the origin of the protein in the PanVax strain. A subclade containing close relatives of the expected A/Puerto Rico/08/1934 strain was identified with only 67% probability. The matrix M1 protein was also identified as type A (Additional file 2: Figure S2). However, further localisation was achieved with less confidence. Uncertainty in its identification is due in part to the high sequence conservation of the M1 protein [29]. In addition, a lower number of mass values were matched to the M1 compared to the NP.

The analysis was repeated using differing combinations of the data and the probabilities of the expected identities were determined as shown in Table 1. The posterior probabilities, shown as percentages, were estimated with FluShuffle and summed over the clade.

The FluResort algorithm was used to determine the possibility of reassortment based on predicted protein identities. For simplicity, the analysis was limited to those that were confidently identified (i.e. proteins HA, NA, NP and M1). All vaccine strains were banned to avoid a trivial match to the PanVax strain itself. The threshold value for reassortment of 4 proteins is 2√4 = 4 composite Z-score units (see equation 5). The composite Z-score without reassortment was 39 units greater than that of the fully reassorted combination (Table 2). This is far greater than the threshold value, which indicates that the non-reassorted combination is a poor fit to the predicted identities. In contrast, no major differences were observed between the composite Z-scores for the fully reassorted combination and those for combinations with 1 or more reassortment events. This favours a single reassortment event, consistent with the expected nature of the PanVax strain.

**Table 2:** Reassortment events in the predicted strain origins for the viral proteins of the PanVax vaccine strain

**Reassortment events**	**Viral antigen**	**Strain**	**Z-score**	**Composite**
0	HA	A/Puerto Rico/08/1934 (H1N1)	21.2	39.6
	M1	A/Puerto Rico/08/1934 (H1N1)	0	
	NA	A/Puerto Rico/08/1934 (H1N1)	18.4	
	NP	A/Puerto Rico/08/1934 (H1N1)	0	
1	HA	A/California/07/2009 (H1N1)	0	0.3
	M1	A/Puerto Rico/08/1934 (H1N1)	0	
	NA	A/California/07/2009 (H1N1)	0.3	
	NP	A/Puerto Rico/08/1934 (H1N1)	0	
2	HA	A/California/07/2009 (H1N1)	0	0.0
	M1	A/Puerto Rico/08/1934 (H1N1)	0	
	NA	A/Louisiana/05/2009 (H1N1)	0	
	NP	A/Puerto Rico/08/1934 (H1N1)	0	

The lowest composite Z-score and the corresponding combination of strains is shown for each number of reassortment events.

### Analysis of seasonal type A strain

A type A strain representative of seasonal H1N1 strains responsible for annual epidemics in human populations during the period 2006–2008 period was analyzed.

**Figure 5:**
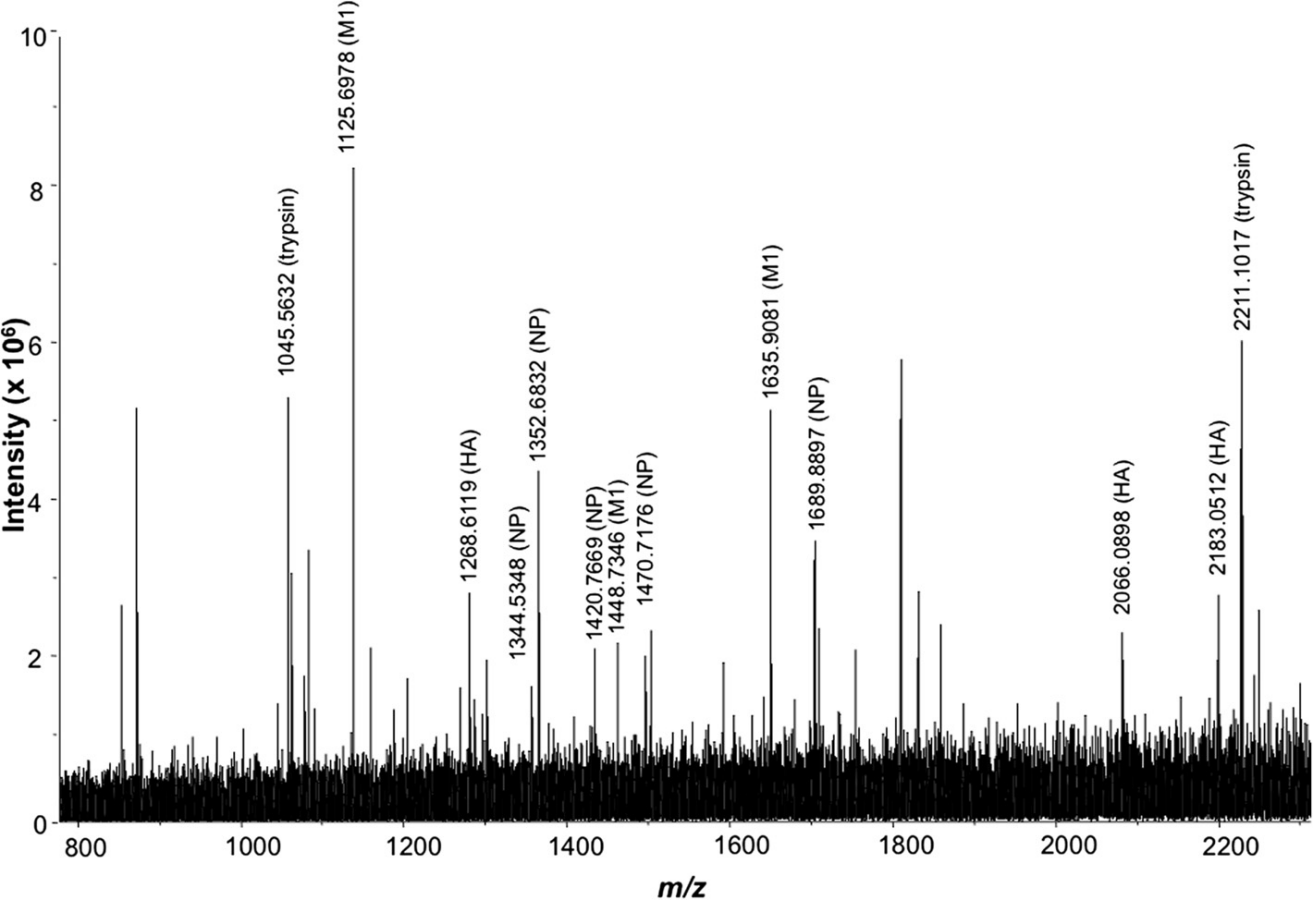
**High resolution MALDI mass spectrum of the tryptic whole virus digest of the A/Solomon Islands/03/2006 strain.** Peaks labelled trypsin denote autolysis products.

The FluShuffle algorithm was used to analyze the high resolution mass spectrum of the tryptic whole virus digest of the A/Solomon Islands/03/2006 strain (Figure 5). Monoisotopic mass values for 14 ion peaks were identified from the mass spectrum after filtering and deisotoping. Most high intensity ions were matched by FluShuffle to theoretical peptide masses from the proteolysis of the major viral proteins. Segments of the HA and M1 proteins were matched to 3 ions whereas peptides derived from the NP matched to 5 ions (Table 3).

**Table 3:** Reassortment events in the predicted identities for the seasonal type A/Solomon Islands/03/2006 strain

**Reassortment events**	**Viral antigen**	**Number of matched tryptic peptides**	**Strain**	**Z-score**	**Composite**
0	HA	3	A/Albany/4835/1948 (H1N1)	1.5	2.6
	M1	3	A/Albany/4835/1948 (H1N1)	0.2	
	NP	5	A/Albany/4835/1948 (H1N1)	0.9	
1	HA	3	A/England/494/2006 (H1N1)	0	0.6
	M1	3	A/Hemsbury/1948 (H1N1)	0.2	
	NP	5	A/Hemsbury/1948 (H1N1)	0.4	
2	HA	3	A/England/494/2006 (H1N1)	0	0.0
	M1	3	A/mallard/Ohio/66/1999 (H1N1)	0	
	NP	5	A/New Jersey/1976 (H1N1)	0	

The lowest composite Z-score and the corresponding combination of strains is shown for each number of reassortment events.

**Figure 6:**
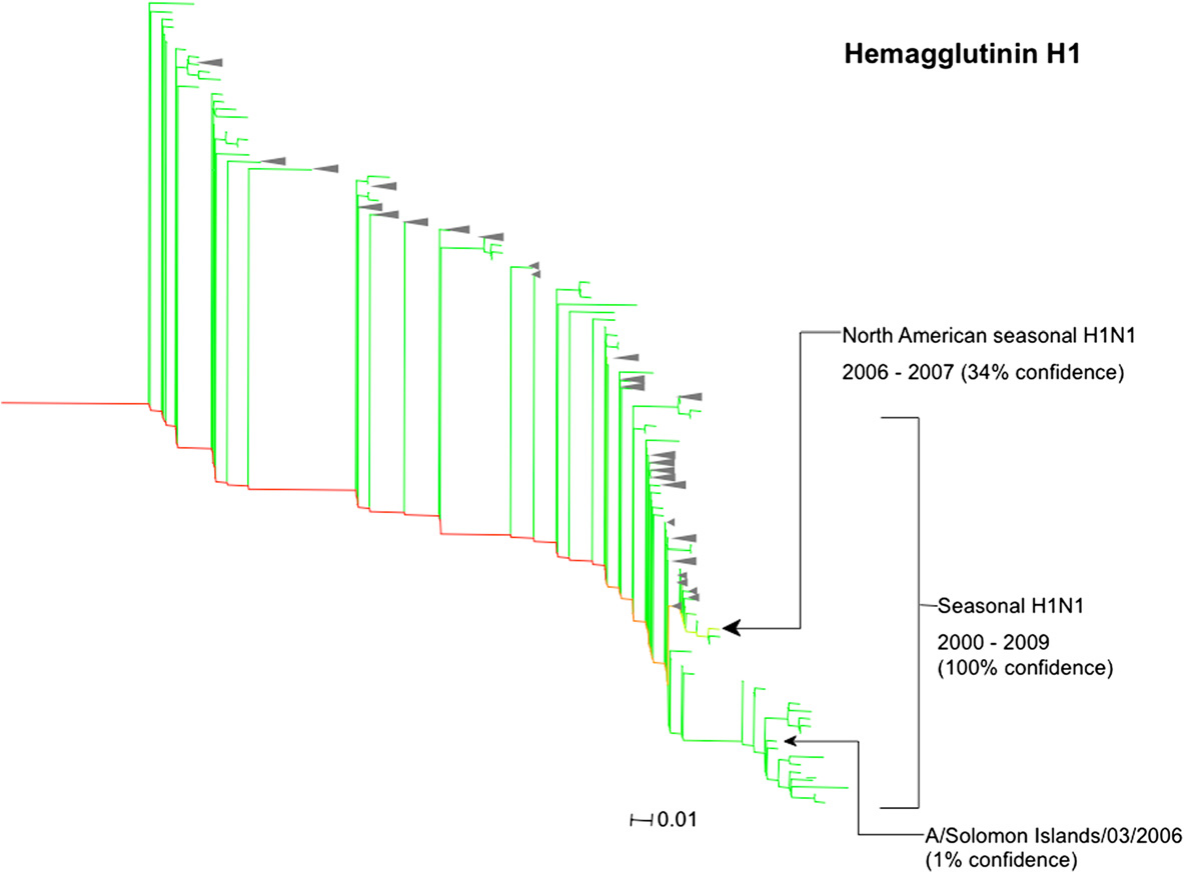
**Phylogenetic tree for the hemagglutinin protein (H1 subtype) with colouration of its predicted identity within the type A/Solomon Islands/03/2006 strain.** The location of the expected identity is labelled and the sum of probabilities for its clade of close relatives is shown in brackets as a percentage. The clade of closely related sequences with the greatest sum probability is marked in bold. The clade containing seasonal H1N1 strains is also shown with its sum of probabilities.

The HA protein was identified to a clade containing seasonal H1N1 strains from 2000 to 2009 with 100% confidence (Figure 6). This is consistent with the identity of the A/Solomon Islands/03/2006 strain. However, further localisation could not be achieved with high confidence (i.e. over 90%). The subclade containing the close relatives of this expected strain was identified with only 1% confidence. The highest probability subclade contained North American seasonal strains from 2006 to 2007 and was identified with 34% confidence. HA protein sequences from these two subclades share 98% sequence identity. This high sequence conservation and the low number of matching peptides prevented the A/Solomon Islands/03/2006 strain from being identified.

The nucleoprotein and matrix M1 proteins were identified as type A with 100% certainty. In the case of the nucleoprotein, localisation was achieved with 90% confidence to a clade containing strains of mixed subtype that contained the expected A/Solomon Islands/03/2006 strain. However, a sub-clade containing the close relatives of the matrix M1 protein of the A/Solomon Islands/03/2006 strain from was identified with only 10% certainty. Once again, different strains could not be distinguished due to only 3 matched ions for this protein and the high sequence conservation observed across strains for the M1 protein.

The predicted identities were analyzed by the FluResort algorithm to determine whether the A/Solomon Islands/03/2006 strain was reassorted. The threshold of reassortment in 3 proteins is approximately 3.4 whereas the maximum decrease in the composite Z-score observed is 2.6 (Table 3). This identifies that the strain is produced without reassortment.

### Analysis of seasonal type B strain

The B/Florida/07/2004 strain is a type B human influenza strain from the Yamagata 88-like lineage. A closely related strain was in circulation until 2009 and formed the basis of the seasonal influenza vaccine in 2008 to 2009 [30].

The FluShuffle algorithm was used to perform a combined analysis on the high resolution mass spectra recorded for whole virus digests of the B/Florida/07/2004 strain with endoproteinases trypsin and Glu-C. Monoisotopic mass values for 25 and 14 peptide ion peaks were identified in the mass spectrum of the tryptic and Glu-C whole virus digests respectively (data not shown). Segments of the hemagglutinin protein were matched to a combined 7 peptide ion mass values in the combined spectral data whereas the nucleoprotein was matched to only 2 peptide ion mass values in the tryptic digest spectrum only. The matrix M1 protein was matched to 2 peptide ion mass values in this spectrum (Table 4).

**Table 4:** Reassortment events in the predicted identities for the seasonal type B/Florida/07/2004 strain

**Reassortment events**	**Viral antigen**	**Number of matched proteolytic peptides**	**Strain**	**Z-score**	**Composite**
0	HA	7	B/Cheongju/437/2008	0	0.76
	M1	2	B/Cheongju/437/2008	0.74	
	NP	2	B/Cheongju/437/2008	0.02	
1	HA	7	B/Cheongju/437/2008	0	0.02
	M1	2	A/chicken/Taiwan/0705/99 (H6N1)	0	
	NP	2	B/Cheongju/437/2008	0.02	
2	HA	7	B/California/15/2007	0	0.0
	M1	2	A/chicken/Taiwan/0705/99 (H6N1)	0	
	NP	2	B/Mie/01/1993	0	

The lowest composite Z-score and the corresponding combination of strains is shown for each number of reassortment events.

The hemagglutinin protein was identified as originating from the Yamagata 88-like lineage of the influenza type B strains with 100% certainty (Additional file 3: Figure S3). The origin of the hemagglutinin protein was further localised to a clade containing close relatives of the B/Florida/07/2004 strain with 100% confidence. The nucleoprotein was identified as originating from a type B strain with 100% certainty, though without further localization, while the matrix M1 protein protein was identified as influenza type B with a confidence of 33% due the detection of few ions associated with it in the tryptic digest spectrum.

Despite the poorer quality of the mass spectral data for this strain, the FluResort algorithm was utilized to determine whether the B/Florida/07/2004 strain was reassorted based on the identities of the HA, NP and M1 proteins. The threshold of reassortment in 3 proteins is again approximately 3.4, whereas the maximum decrease in the composite Z-score in Table 4 is 0.76. This small decrease is consistent with a lack of reassortment in the generation of the type B/Florida/07/2004 strain. The predicted non-reassortant strain type B/Cheongjuj/437/2008 has 99% sequence identity to the type B/Florida/07/2004 strain across the HA, M1 and NP viral proteins.

### Testing of FluShuffle and FluResort algorithms with simulated mass spectral data

The performance of the FluShuffle algorithm was evaluated more extensively through the analysis of 500 mass spectral datasets for simulated whole virus digests both in the Markov chain Monte Carlo (MCMC) [38] or single-protein modes. The former resulted in significant decreases in the proportion of misidentified viral subtypes (data not shown). The proportion of correct identifications increased with increasing sequence coverage since increasing the amount of mass spectral data improves the confidence of protein identification. No changes were observed with increased numbers of noise or background ion peaks. This is due to the high resolution and mass accuracy offered by the MALDI FT-ICR instrument in which ions of very similar mass to charge can be easily resolved, and random noise peaks in the spectrum are unlikely to match to those of a viral protein-derived peptide.

## Methods

### Influenza strains

The PanVax H1N1 vaccine was donated by CSL Biotherapies, CSL Limited (Parkville, Victoria, Australia) and used without further purification. The vaccine contains the NYMC X-181 strain at a dose of 30 ng/mL of HA. The genomic segments encoding the HA, NA and PB1 proteins in the vaccine strain are derived from the pandemic H1N1 2009 strain A/California/07/2009. The other segments are derived from NYMC X-157, a lab-modified A/Puerto Rico/08/1934 strain. Accession numbers used for the expected viral protein identities were GenBank: ACP44189 (HA), ACT36688 (NA), ACF41835 (M1) and ABD77679 (NP).

Strains B/Florida/07/2004 and A/Solomon Islands/03/2006 were obtained from Advanced ImmunoChemicals Inc. (Long Beach, CA, USA) as inactivated virus preparations from egg allantoic fluid. Strain B/Florida/07/2004 is a type B virus from the Yamagata 88-like lineage. The accessions used for the expected protein identities were GenBank: ACF54213 (HA), ACF54217 (NA), ACF54214 (M1) and ACF54218 (NP). Strain A/Solomon Islands/03/2006 is a seasonal virus with a H1N1 subtype. Accessions used for the expected viral protein identities were GenBank: ABU99109 (HA), ABU99068 (NA), ACD37437 (M1) and ACX46205 (NP).

### Whole virus digest of influenza strains

A suspension corresponding to 3 μg of whole virus was concentrated to near dryness and resolubilized in 50 μL digestion buffer (50 mM NH_4_HCO_3_, 10% acetonitrile and 2 mM dithiothreitol, pH 7.8). Sequencing grade porcine trypsin (Promega Corporation, WI, USA) or Glu-C from *Staphylococcus aureus V8* (Roche Diagnostics GmbH, Mannheim, Germany) was added at 6–14 ng/μL and the solution was incubated overnight at 37°C and 25°C respectively.

### High resolution MALDI FT-ICR mass spectrometry

Each digest solution was added to five volumes of MALDI matrix (5 mg/mL α-cyano-4-hydroxycinnaminic acid, 50% acetonitrile, 0.1% TFA), spotted onto a MALDI sample plate (MTP AnchorChip™400/384 TF, Bruker Daltonics, Billerica, MA, USA) and air-dried at room temperature. MALDI FT-ICR mass spectra were recorded on a 7T Bruker APEX-Qe mass spectrometer (Bruker Daltonics, Billerica, MA, USA) in the positive ion mode using a 35% laser power as previously described [27]. Raw peak lists were converted to MS1 format [40] and monoisotopic values were identified using Hardklör [41] with a maximum charge state of +1 and no centroiding.

### Viral protein identification from mass spectra

Peptide library files and the annotation library file were produced by PepGen. FluShuffle was used to identify viral proteins from the experimental peak lists of monoisotopic mass values. Analysis of peak lists was performed with consideration for variable methionine oxidation, variable pyroglutamate formation, a maximum of 1 missed cleavage and a mass tolerance of 5 ppm. Matches for both trypsin and endoproteinase Glu-C digests were simultaneously incorporated into the calculation of the posterior probability during Gibbs sampling. The information from multiple digests was integrated into one prediction for the viral protein identity.

Predicted identities were plotted onto phylogenetic trees that were visualised using the Archeopteryx software. Phylogenetic trees were produced with FastTree 2.0 [42] using from non-redundant viral protein sequences from NCBI and aligned with MUSCLE [43]. Trees generated were mid-point rooted to increase the interpretability of branch distances. FluResort was used to establish whether or not the virus was reassorted. Laboratory produced strains were banned from the analysis to avoid the trivial detection of artificially reassorted strains. A minimum difference of 2√N in composite Z-scores was required to define reassortment.

### Simulation of large numbers of datasets to test algorithms with FluSim

Simulated peak lists were constructed for analysis by FluShuffle to evaluate its performance in predicting protein of known identities. FluSim generates random peak lists from the monoisotopic mass value for proteolytic peptide ions generated *in silico* from viral proteins and contaminant protein sequences. 500 random peak lists resulting from a simulated tryptic digest of viral protein sequences were generated using FluSim utilizing 5-20% sequence coverage with the addition of 20% spurious noise peaks, 0.1% contaminant coverage, variable methionine oxidation, variable pyroglutamate formation, a maximum of 1 missed cleavage and a mass tolerance of 5 ppm.

One accession was randomly selected for each protein. Each accession was also associated with a set of peptide masses resulting from proteolytic digestion. A subset of the peptide ion masses was then randomly picked for inclusion into the peak list such that the specified sequence coverage was achieved. Noise peaks were then added at random across the mass acquisition range to the peak list. The peptide set for contaminants was also collated and contaminant masses were added according to the specified contaminant coverage. A random mass error was added to each *m/z* value within a specified mass tolerance.

FluShuffle was used to establish the expected protein identities as described above. A correct identification is defined as a predicted identity that is less than 0.05 substitutions per site from the expected identity.

## Conclusions

The FluShuffle and FluResort algorithms correctly identified the reassorted nature of the PanVax strain and the identity of the viral proteins that comprise it. Both of the seasonal strains studied were found not to be reassorted and the clade containing the strain under investigation was identified with 100% confidence in terms of the hemagglutinin protein in both cases, and with less confidence for other proteins where fewer peptides were detected and high sequence conservation exists among strains. In the case of highly related strains, with similar protein sequences, peptide segments that span regions of sequence difference must be detected in the mass spectrum in order for strains to be differentiated from one another. As peptides spanning the entire sequence are usually not detected, particularly in the mass spectra of multiple viral proteins from whole virus digests, the identification of a single strain or a set of strains within a clade may not be possible.

Nonetheless, the algorithms significantly improve the capability of the proteotyping approach to identify reassorted viruses that pose the greatest pandemic risk. The FluShuffle algorithm extends the capabilities of the proteotyping approach, beyond the determination of viral type, subtype or lineage, by allowing the identification of the strain origin of each protein. FluShuffle can also perform a combined analysis of data from multiple proteolytic digests that outperforms the single protein approach common to protein mass mapping or fingerprinting algorithms [39]. FluResort identifies reassortment more rapidly than other algorithms since it determines whether a single virus has reassorted from existing known strains rather than identifying all the reassortment events in the evolutionary history of a viral strain.

## Availability and requirements

**Project name:** FluShuffle and FluResort

**Project home page:**http://sydney.edu.au/science/molecular_bioscience/downard/flushuffle.html

**Operating system:** web-based platform independent

**Programming language:** C++

**License:** Free access for non-commercial use.

## Competing interests

The authors have no competing interests.

## Authors' contributions

KMD conceived the project, oversaw its design, coordination and progression and drafted the manuscript. ATLL and JWHW contributed to the writing of sections of the manuscript. ATLL designed, developed and tested the algorithms with the advice and participation of JWHW. ATLL prepared the virus digests while KMD carried out the mass spectrometry experiments. All authors read and approved the final manuscript.

## Supplementary Material

Additional file 1 **Figure S1.** Phylogenetic tree for the neuraminidase protein. Click here for file

Additional file 2 **Figure S2.** Phylogenetic tree for the matrix M1 protein. Click here for file

Additional file 3 **Figure S3.** Phylogenetic tree for the hemagglutinin protein. Click here for file
