# Supplementary figures and images for: FluShuffle and FluResort: new algorithms to identify reassorted strains of the influenza virus by mass spectrometry
Source: BMC Bioinformatics. 2012 Aug 20;13:208. doi: 10.1186/1471-2105-13-208 (PMC3505172; doi:10.1186/1471-2105-13-208)

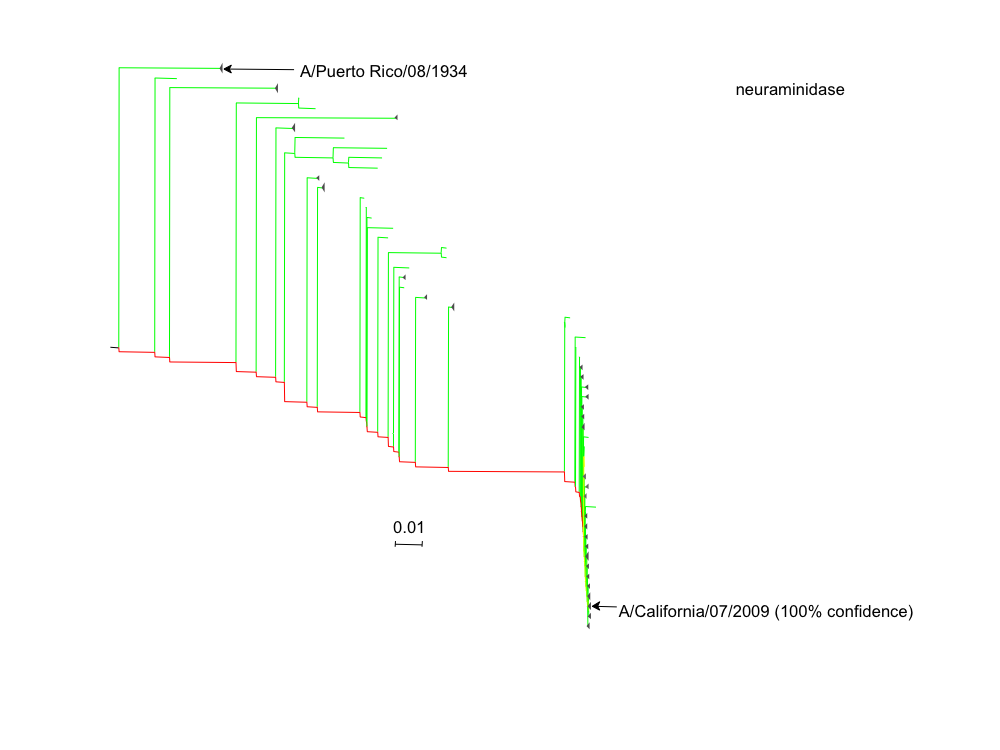

Supplement: Additional file 1 — Figure S1. Phylogenetic tree for the neuraminidase protein. [file 1471-2105-13-208-S1.tiff]

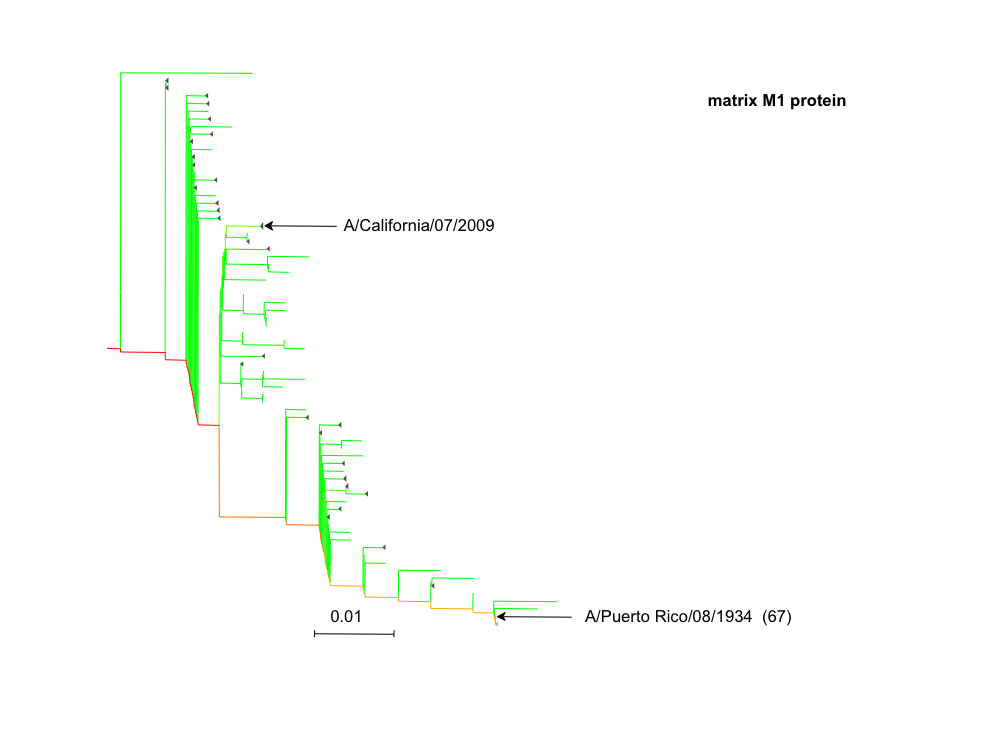

Supplement: Additional file 2 — Figure S2. Phylogenetic tree for the matrix M1 protein. [file 1471-2105-13-208-S2.tiff]

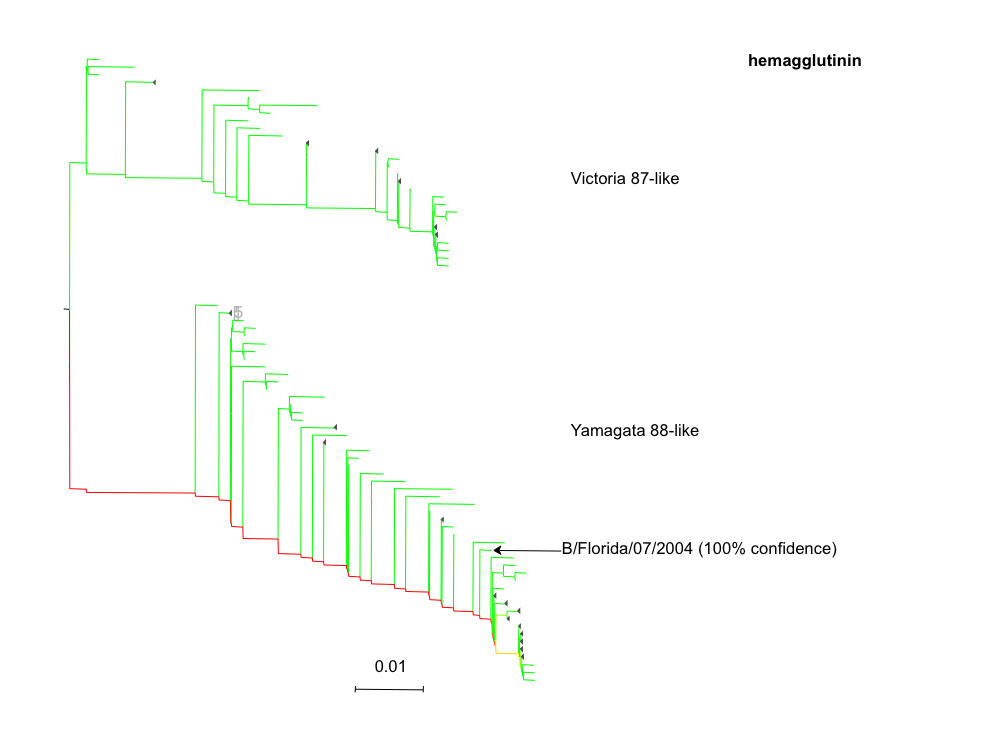

Supplement: Additional file 3 — Figure S3. Phylogenetic tree for the hemagglutinin protein. [file 1471-2105-13-208-S3.tiff]
